# Efficacy of Platelet-Rich Fibrin in Treatment of Multiple Adjacent Gingival Recession Defects Using Minimally Invasive Coronally Advanced Flap and Modified Coronally Advanced Flap: A Split-Mouth Randomized Controlled Trial

**DOI:** 10.1155/ijod/5585609

**Published:** 2025-07-29

**Authors:** Vandana Daga, Ashita Uppoor, Sangeeta Umesh Nayak, Priyanka Paramita Sahu, David Kadakampally

**Affiliations:** Department of Periodontology, Manipal College of Dental Sciences Mangalore, Manipal Academy of Higher Education, Manipal 576104, Karnataka, India

**Keywords:** gingival recession, minimally invasive surgical procedures, platelet-rich fibrin, randomized controlled trial, surgical flaps

## Abstract

**Background:** The split-mouth randomized clinical trial evaluated and compared the clinical efficacy and patient-centered outcomes of platelet-rich fibrin (PRF) combined with modified coronally advanced flap (MCAF) and minimally invasive coronally advanced flap (MICAF) techniques for the treatment of multiple adjacent gingival recession (GR) Cairo's RT1 and/or RT2.

**Methods:** Eighteen participants with multiple GR defects were treated using MCAF + PRF and MICAF + PRF approaches in a split-mouth design. A qualified periodontist examined the periodontal clinical parameters (recession height [RH] recession width [RW], clinical attachment level [CAL] gain, probing depth [PD], keratinized tissue width [KTW], and gingival thickness [GT]) and aesthetics at baseline and 6 months later. After 7 days, 3 months, and 6 months, patient-centered outcomes relating to pain/discomfort and aesthetics were evaluated using a visual analog scale (VAS) and a questionnaire. A statistical study was performed on the variations between the original recording and the subsequent months.

**Results:** Both the surgical methods showed statistically significant improvements in all clinical parameters, such as RH, RW, CAL, PD, and KTW (*p* < 0.001). MICAF + PRF was found to have a similar clinical efficacy to MCAF + PRF for tissue gain; yet between-group differences in GT after 6 months were not statistically significant. Patient-related outcome measures (PROMs) were significantly better in MICAF + PRF with lower VAS pain scores (*p* < 0.001) and higher ratings of esthetic satisfaction (*p* < 0.001) at 6 months' follow-up.

**Trial Registration:** Name of the Registry: Clinical Trials Registry - India (CTRI); Registration Number: CTRI/2019/01/016994

**Summary**

• MICAF yields notable results in gingival recession (GR) with high patient acceptance

## 1. Introduction

Gingival recession (GR) defect treatment is the most common challenge in periodontics. GR, besides its esthetic implications, increases the chances of root sensitivity and cavities. The presence of exposed roots complicates proper dental care. Numerous therapeutic techniques for managing GR defects have been documented as successful in the literature. According to evidence-based periodontics, coronally advanced flap (CAF) with connective tissue graft (CTG) is the gold standard for treating GR [1–3]. This technique for treating multiple GR defects has limited clinical applicability due to the requirement for a second surgical site and inadequate graft availability [4–6].

A periodontist's primary objective is to achieve complete root coverage (CRC), while minimizing patient morbidity. In the current period of personalized periodontics, patient-reported outcome measures (PROMs) significantly influence the choice of surgical techniques and determine the success of perioplastic surgery. Researchers and clinicians worldwide are persistently innovating minimally invasive, patient-friendly surgical procedures. Since 1990, minimally invasive dentistry has made significant strides in the realm of perioplastic surgeries, marked by numerous innovations.

This minimally invasive technique, vestibular incision subperiosteal tunnel approach (VISTA), has demonstrated effective results for treating various GR abnormalities. A longer vestibular incision and a subperiosteal tunnel design impaired blood supply and delayed healing. In 2018, the minimally invasive coronally advanced flap (MICAF), a modification of VISTA, was developed for addressing various GR anomalies. The combined procedure involves making a shorter vertical incision in the connected gingiva instead of a longer access incision in the vestibule, utilizing both supra and subperiosteal tunnel designs. The MICAF exhibits an apical subperiosteal tunnel that corresponds to the recession height (RH) but is subperiosteal only in its coronal aspect. Ensuring sufficient blood flow minimizes postoperative complications [7].

Rajendran et al. [7] study compared MICAF and modified coronally advanced flap (MCAF) for treating multiple GR abnormalities. The root coverage was successful in both surgical procedures, yet there was no enhancement in KTW and GT. Studies suggest that grafting during root coverage improves long-term clinical results and stability of the gingival margin. Systematic reviews and meta-analyses have extensively studied and confirmed platelet-rich fibrin (PRF) as an effective alternative to CTG for graft materials [7]. Since its introduction by Zuchelli and De Sanctis [8] in 2009, MCAF has gained popularity as a preferred approach for managing multiple adjacent GR defects due to its vertical incision-free flap design. Reducing scar formation probability during healing improved esthetic outcomes [8]. Therefore, this study compared MCAF + PRF and MICAF + PRF, both clinically and with patient-reported outcomes, for the treatment of multiple adjacent GR defects.

## 2. Materials and Methods

This trial, a split-mouth, randomized study, was led by the Department of Periodontics at MCODS, MAHE, India. In total, 18 individuals (11 men and 7 women), who possessed a total of 77 recession abnormalities (Cairo's RT1 or 2), were selected for this study. The patient received MICAF + PRF treatment on one side and MCAF + PRF treatment on the other side. The study received ethical approval from the Human Subject Ethics Board of MCODS, Mangalore, MAHE, in accordance with the 2000 revision of the 1975 Helsinki Declaration.

The study included participants with two or more adjacent maxillary/mandibular defects of Cairo's RT1 or 2, a difference in GR depth and clinical attachment ≤1 and 2 mm, respectively, a gingival biotype <1 mm, probing depth (PD) <3 mm, full mouth plaque and gingival index (GI) score <20%, and no tooth malpositioning or cervical abrasions treatment. Patients with systemic complications, invasive periodontal procedures in the last 2 years, smoking status, pregnancy or lactation, fixed orthodontic and removable appliances, erratic dental/maintenance habits, and poor plaque control were excluded.

### 2.1. Sample Size Calculation

Sample size was calculated using G^*⁣*^*∗*^^ power software for two-sided paired *t* test [9]. Based on the study by Rajendran et al. [7], at 5% level of significance and 90% power, a sample size of 18 was required to achieve the detection of a minimum clinically relevant difference [7].

### 2.2. Randomization and Blinding

Participants were randomly assigned to either the MICAF or MCAF group through simple randomization by tossing a coin. A coin was tossed for every eligible participant by an independent researcher who did not take part in the intervention or outcome measurement, “heads” assigned the participant to the MICAF group and “tails” to the MCAF group. This process allowed every participant an equal and unbiased chance of being allocated. Nonetheless, since a coin toss does not inherently ensure allocation concealment, the assignment to groups was revealed after enrollment of participants in order to limit selection bias. The operation site (left or right) was decided through another coin toss that was independent of the treatment allocation. Once the participant had been randomized into either the MICAF or MCAF group, a second coin toss was undertaken: “heads” signified that the procedure would be carried out on the right side and “tails” on the left side. This guaranteed that the surgical side was randomly allocated and not biased by operator preference or anatomical considerations, thus avoiding selection bias for site assignment.

Blinding was accomplished by masking the participants as to the type of surgery they had received. To ensure an identical postoperative appearance and reduce identification, the MICAF and MCAF groups received the same clinical care, including the use of orthodontic buttons and periodontal packs. DK and SN, the outcome assessors, were not involved in the intervention or randomization and were blinded to the group assignments. Since the surgeon Vandana Daga needed to know the allocated procedure to perform the surgery, she was not blinded. Additionally, Ashita Uppoor, who supervised the procedures with scheduling and group allocation, was not blinded, but was blinded to assessment of outcomes.

### 2.3. Initial Therapy and Clinical Measurements

Nonsurgical periodontal therapy comprising scaling and root planing and oral hygiene instructions was given to all the participants. Surgical procedures for management of GR defects were performed only after the patient could maintain his/her oral hygiene.

The parameters recorded at baseline and 6-month follow-up were as follows:

1. RH: Distance from the cementoenamel junction to the free gingival margin.
2. Recession width (RW): Distance between the mesial and distal gingival margins, 1 mm apical to the CEJ.
3. PD: Distance from gingival margin to the base of the gingival sulcus.
4. Clinical attachment level: Summation of RH and PD.
5. Width of keratinized tissue (WKT): Distance from the most apical point of the gingival margin to the mucogingival junction (MGJ).
6. Gingival thickness (GT): Measured by inserting an endodontic reamer (with a silicone rubber stopper) into the gingival/mucosal margin at the attached gingiva or alveolar mucosa at a 90° angle to the long axis of the tooth and then measuring the distance from the tip of the reamer to the inner surface of the silicone stopper with a graduated scale.
7. Maximum root coverage level (MRCL): Introduced by Zuchelli et al. [10] for predetermining the line of root coverage in GR defects with noncarious cervical lesions (NCCLs) where detection of CEJ is difficult.
8. Root coverage esthetic score (RES): Used to assess esthetic outcomes at 6-month postoperative visit and is evaluated based upon five variables-level of the gingival margin, marginal tissue contour, soft tissue texture, MGJ alignment, and gingival color. The maximum score was 10, with CRC, mean root coverage (MRC), and other four variables being scored as 6, 3, and 1 each, respectively [11].

### 2.4. Patient Evaluation and Postoperative Morbidity

A VAS and questionnaire were used to assess patient-reported outcomes. Postoperative morbidity and patient satisfaction were evaluated based on treatment outcomes using the visual analog scale (VAS). Postsurgery of 1 week, the participants rated their postoperative courses on a 0–100 scale, with 0 being very bad, 50 average, and 100 excellent. Dentinal sensitivity was measured using the same scale at baseline, 3 months, and 6 months after surgery. At the 3-month and 6-month marks postsurgery, patients reported both adverse effects and esthetic outcomes. The scoring was consistent, based on color contrast, root coverage, and overall satisfaction.

### 2.5. Surgical Procedure and Postoperative Instructions

After the local anesthesia was administered, curettes were used to remove debris, biofilm, and calculus from the exposed root surface and likely depths. The orthodontic buttons were attached to the teeth before the soft tissue surgery.

**Figure 1:**
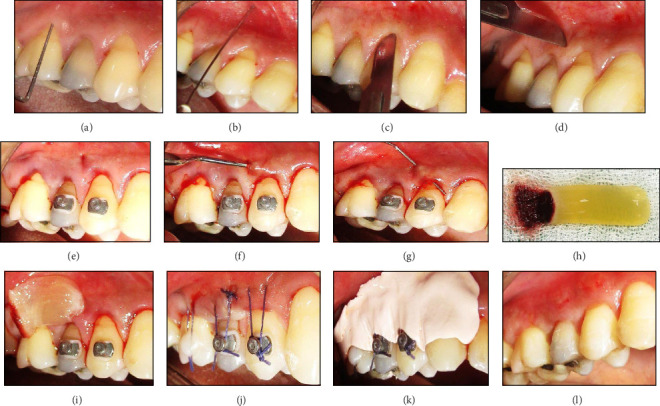
(a-l) Group 1 MICAF + PRF. (a) Preoperative measurement of gingival recession using a periodontal probe. (b) Administration of local anesthesia. (c-d) Incision design to create a minimally invasive coronally advanced flap (MICAF). (e–g) Preparation of a tunnel. (h) Prepared PRF membrane obtained after centrifugation. (i) Stabilization of the PRF over the defect. (j) Suturing of the flap to secure the PRF and achieve tension-free closure. (k) Postoperative periodontal dressing for site protection. (l) Healed surgical site showing improved gingival coverage and integration.

In the MICAF + PRF group, a 15c BP blade was used for sulcular incisions, while a small vertical incision was made on the adjacent attached gingiva [7]. A microsurgical periosteal elevator was used to create a subperiosteal tunnel through the sulcular incisions, down to a depth equivalent to RH. Through the vertical incision, a supraperiosteal tunnel was created using a tunneling knife. The MGJ tunnel was extended and merged coronally with the subperiosteal tunnel. The flap was handled carefully before being installed in the defect site using PRF prepared with Choukroun's method [12]. The soft tissue complex was coronally displaced and sutured in its entirety. The orthodontic buttons secured the sutures for increased anchorage, and a simple interrupted suture was utilized for the vertical incision (Figure 1a–l, Group 1 MICAF + PRF).

**Figure 2:**
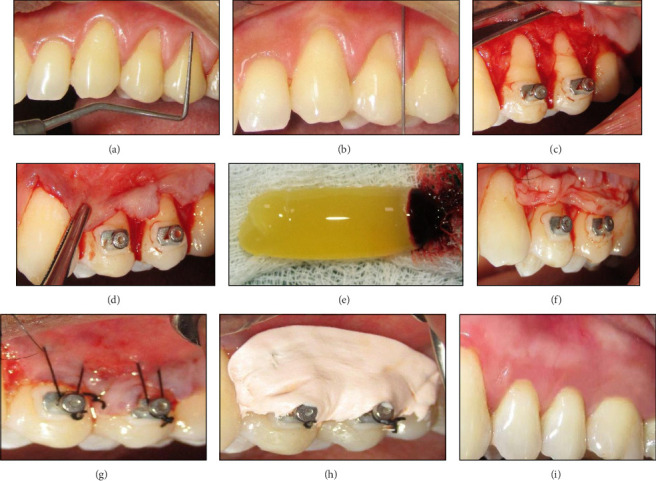
(a–i) Group 2 MCAF + PRF. (a) Preoperative measurement of gingival recession using a periodontal probe. (b) Administration of local anesthesia. (c) Flap reflection showcasing root exposure and defect preparation. (d) Tension-free advancement of the flap. (e) Prepared PRF membrane after centrifugation. (f): PRF secured over the defects with flap repositioning. (g) Suturing of the flap to stabilize the graft and PRF in position. (h): Postoperative dressing applied to protect the surgical site. (i) Healed surgical site showing improved gingival coverage and integration.

Similarly, in the MCAF + PRF group, sulcular incisions, and submarginal oblique horizontal incisions were extended one tooth beyond the defect site both mesially and distally [10]. This led to the formation of a full-thickness mucoperiosteal flap that was reflected beyond the MGJ to expose 3–4 mm of healthy bone, and a split-thickness flap was created beyond the MGJ. No vertical incisions were incorporated in this type of flap design. PRF was prepared using Choukroun's protocol and placed in the defect site [12]. The flap was coronally advanced and sutured over the de-epithelized papillae. Orthodontic buttons were held in place by suturing them around (Figure 2a–i: Group 2—MCAF + PRF).

The 5–0 silk sutures were used, and periodontal dressing was placed in procedures where the gingival margin was coronal to the CEJ for both MICAF + PRF and MCAF + PRF. About 0.2% chlorhexidine mouthwash should be used by patients to maintain plaque control, while surgical chair time is recorded for all procedures. In the immediate postoperative phase, patients were given analgesics (Tab IMOL PLUS: Ibuprofen [400 mg] + Paracetamol [325 mg] + Caffeine [25 mg]) as needed. After 2 weeks of postsurgery, sutures were removed from patients. Recalled for reevaluation, oral hygiene reinforcement, and oral prophylaxis as needed at 1-, 3-, and 6-month postsurgery.

### 2.6. Statistical Analysis

Statistical analysis was conducted using the statistical package for the social sciences (SPSS) version 24.0 (IBM Corp. Armonk, NY, USA). Descriptive statistics, including means and standard deviations, were calculated for each clinical parameter. For between-group comparisons (MICAF vs. MCAF), the Mann–Whitney *U* test was used to compare differences in continuous nonparametric variables. The Wilcoxon signed rank test was used for preintervention and postintervention comparison within the group. A significance level of *α* = 0.05 was used for all statistical tests. Results with *p* < 0.05 were considered statistically significant.

## 3. Results

All 18 participants completed the study as well as the 6-month follow-up phase, without any dropout, or postoperative complications. A total of 77 sites of labial/buccal GR were included, comprising 39 sites in the MICAF + PRF group and 38 in the MCAF + PRF group.  Table 1 shows the within the group comparison of plaque index (PI) and GI at baseline and 6 months for both groups.

Within the groups, in group 1 (MICAF + PRF), PI scores slightly increased from 0.512 at baseline to 0.567 at the end of 6 months. However, the increase in scores was statistically significant with *p* value = 0.006, suggesting a rise in the plaque level. The mean GI score also increased from baseline 0.288 to 0.363 (6 months), and the change was statistically significant (*p*=0.001), which indicated a slight increase in gingival inflammation in MICAF + PRF. In group 2 (MCAF + PRF), the PI score increased from 0.487 to 0.556 (*p* =0.001), showing plaque accumulation similar to that of group 1. Statistically significant (*p* < 0.001), change in the GI was observed in group 2. GI scores increased from 0.291 to 0.37, indicating gingival inflammation (Tables 1 and 2).

In Group 1, all probing measurements were significantly improved at the 6-month follow-up compared to baseline. RH significantly reduced from 2.54 ± 0.50 mm to 0.06 ± 0.13 mm, representing a mean difference of 2.48 ± 0.45 mm (*p* < 0.001), with near CRC. RW also reduced significantly (*p* < 0.001). PD reduced significantly from baseline at 2.48 ± 0.37 mm to 6 months at 1.87 ± 0.23 mm, representing a mean decrease of 0.61 ± 0.33 mm (p< 0.001). The CAL significantly improved, with values reduced from 5.04 ± 0.75 mm to 1.93 ± 0.29 mm (*p* < 0.001). The WKT and GT also improved (*p* < 0.001). These results together show that the MICAF + PRF procedure was clinically effective in achieving periodontal tissue improvements that were both clinically significant and statistically significant over the course of the study (Table 3).

In Group 2, all clinical probing measurements were significantly improved from baseline to 6-month follow-up. RH reduced significantly from 2.52 ± 0.51 mm to 0.07 ± 0.14 mm with a mean decrease of 2.44 ± 0.51 mm (*p* < 0.001), reflecting extensive root coverage. RW also improved, decreasing with a mean difference of 2.69 ± 0.53 mm (*p* < 0.001). Probing PD decreased from 2.44 ± 0.38 mm to 1.87 ± 0.23 mm, (*p* < 0.001). CAL demonstrated significant improvement from 4.98 ± 0.79 mm at baseline to 1.94 ± 0.31 mm at 6 months (*p* < 0.001). The WKT significantly improved (*p* < 0.001), indicating increased soft tissue stability. GT also significantly improved, rising from 1.02 ± 0.08 mm to 1.22 ± 0.26 mm, with a mean difference of 0.20 ± 0.26 mm (*p*=0.004). These results indicate that the MCAF + PRF procedure led to significant and clinically relevant improvements in all periodontal parameters assessed during the study period (Table 4).

The comparison between GI and PI at both baseline and at 6 months showed that there were no statistically significant differences between Group 1 and Group 2. At baseline, the mean PI scores were 0.5125 and 0.4875 for Group 1 and Group 2, respectively (*p*=0.405). At 6 months follow-up, the mean PI scores were 0.5675 and 0.55625 for Groups 1 and 2, respectively (*p*=0.79). The difference in PI over time (PI DIFF) was also not significantly different between the groups (*p*=0.49). Likewise, the mean GI at baseline was 0.28875 in Group 1 and 0.29125 in Group 2 (*p*=0.924), whereas at 6 months, the GI was 0.36375 and 0.37375, respectively (*p*=0.758). The difference in GI from baseline to follow-up (GI DIFF) was again statistically not significant between the groups (*p*=0.704).

PROMs showed statistically significant differences between the two treatments. Group 1 reported better patient satisfaction scores, lower scores for postoperative pain, and dentinal hypersensitivity (*p*  < 0.001). Moreover, group 1 patients reported more favorable esthetic satisfaction and fewer postoperative complications such as pain, bleeding, and discomfort. The MICAF + PRF group reported statistically significant superior outcomes in terms of patient satisfaction (*p*  < 0.001). These results highlight the clinical acceptability and patient preference for the MICAF + PRF procedure.

## 4. Discussion

This study aimed to compare the clinical and patient-reported outcomes of MICAF + PRF and MCAF + PRF in treating multiple adjacent GR defects. The study focused on the GR defects Cairo's RT 1 and 2. At the 6-month follow-up, MICAF + PRF and MCAF + PRF significantly decreased RH, RW, PD, and increased CAL, KTW, and GT compared to baseline. The clinical outcomes for VISTA + PRF and CAF + PRF in the treatment of multiple GR defects are consistent with earlier research findings.

Placing the gingival margin coronal to the CEJ significantly contributes to the longevity of the coronally repositioned soft tissue flap. In our current investigation, the gingival margin was repositioned coronally to the CEJ, and both groups utilized orthodontic buttons, as demonstrated in a split-mouth RCT. Orthodontic buttons were used to enhance anchorage and stability for the relocated flap margin.

Graziani et al. [13] determined in a meta-analysis that no single surgical technique excels for addressing multiple GR defects. Modifications to flap design or the use of graft biomaterial can improve root coverage outcomes.

Patients' increasing preference for minimal postoperative morbidity and heightened esthetic expectations has led to the development and utilization of various CTG alternatives like PRF, alloderm, amnion, and chorion membrane for treating GR defects [14]. New research reveals promising outcomes in treating multiple GRs with PRF [15]. Multiple systematic reviews and meta-analyses confirmed the positive impact of PRF on root coverage procedure results [16–18].

Patients prefer PRF over CTG because it eliminates the need for a second surgical site and related complications. This compound can be acquired in sufficient amounts and effortlessly shaped for addressing several GR issues. Extensive research has been conducted on PRF's potential for reducing surgical time and maintaining clinician ergonomics through soft tissue regeneration. PRF's angiogenic potential is attributed to the release of PDGF, VEGF, and TGF-β1, while its ability to prevent infections lies in the fibrous matrix's stimulation of avb3, resulting in enhanced cell binding and the phagocytosis and ROS killing capabilities of neutrophils. Unlike alloderm, amnion, and chorion membrane, PRF does not undergo tissue necrosis [4, 19, 20]. The PRF application in surgical procedures significantly enhances KTW growth at the defect site through stimulation of the overlying keratinized epithelium by the influence of newly formed connective tissue.

The present study led to a GT enhancement in both surgical methods employing PRF, from baseline to 6 months postsurgery. The gain did not reach statistical significance. The systematic review and network meta-analysis revealed that clinicians and patients held varying perceptions regarding successful treatment outcomes[21]. A thick gingival biotype, while beneficial for the longevity of root coverage procedures, will not be considered a success if the patient finds it bulky and unattractive [22].

Patients prioritize the esthetic outcome when considering root coverage treatments for GR. According to several studies, the use of PRF for root coverage results in improved texture and color match due to accelerated healing. At 6 months follow-up, there was no significant difference in RES between the two procedures. The RES of MICAF + PRF was slightly higher than MCAF + PRF in the study. Its minimally invasive flap design and limited tissue manipulation are possibly the reasons for this.

Multiple Cairo's RT1 and 2 GR defects can be treated effectively with both MICAF + PRF and MCAF + PRF. PRF proves effective in root coverage procedures as an alternative graft biomaterial. Patient acceptance was greater with MICAF + PRF. To confirm the results of this 6-month follow-up RCT, long-term studies are necessary.

In the current era of personalized periodontics, a harmonious partnership between patient and clinician is emphasized. A successful perioplastic procedure fulfills both the clinician's and patient's esthetic and functional expectations. The patients' reported outcomes significantly influence the chosen surgical treatment course. In the present study, patient-reported outcome measures were assessed to measure the dentinal hypersensitivity, esthetic satisfaction, postoperative pain, and bleeding. The results obtained revealed a superior and favorable outcome for MICAF + PRF, and it was chosen by the patients as the preferred surgical procedure. The inclusion of PROMs in our study as one of the defining parameters in the decision-making process agrees with a consensus report from the American Academy of Periodontology Regeneration Workshop in 2016 and a systematic review and network meta-analysis by Cairo et al. [6, 23]. A minimally invasive surgical technique leads to improved patient acceptance.

## 5. Conclusion

According to our study, Cairo's RT1 and 2 GR defects were effectively treated with MICAF + PRF and MCAF + PRF, suggesting that PRF can serve as a viable substitute for traditional graft materials in root coverage procedures. Considering patient acceptance, minimal invasiveness, and favorable root coverage outcomes, MICAF + PRF is the preferred choice. More long-term research is needed to confirm the findings of this study.

## Figures and Tables

**Table 1:** Intragroup comparison of plaque and gingival indices at baseline and 6 months.

Procedure	Clinical parameters	Mean	*N*	Std. deviation	Paired differences		*p*-Value
					Mean difference	Std. deviation	
Group 1	PI-B	0.5125	18	0.05825	−0.055	0.040356	**0.006**
	PI 6	0.5675	18	0.087953			
	GI-B	0.28875	18	0.051944	−0.075	0.042088	**0.001**
	GI 6	0.36375	18	0.0563			

Group 2	PI-B	0.4875	18	0.05825	−0.06875	0.037201	**0.001**
	PI 6	0.55625	18	0.077632			
	GI-B	0.29125	18	0.050551	−0.0825	0.034949	**<0.001**
	GI 6	0.37375	18	0.070293			

*Note*: Bold represents *p* values are statistically significant.

Abbreviations: GI 6, gingival index at 6 months; GI-B, gingival index at baseline; PI 6, plaque index at 6 months; PI-B, plaque index at baseline.

**Table 2:** Intergroup comparison of plaque and gingival indices at baseline and 6 months.

Clinical parameters	Procedure	*N*	Mean	Std. deviation	*p*-Value
PI-B months	Group 1	18	0.5125	0.05825	0.405
	Group 2	18	0.4875	0.05825	

PI 6 months	Group 1	18	0.5675	0.087953	0.79
	Group 2	18	0.55625	0.077632	

PI DIFF	Group 1	18	0.055	0.040356	0.49
	Group 2	18	0.06875	0.037201	

GI-B months	Group 1	18	0.28875	0.051944	0.924
	Group 2	18	0.29125	0.050551	

GI 6 months	Group 1	18	0.36375	0.0563	0.758
	Group 2	18	0.37375	0.070293	

GI DIFF	Group 1	18	0.075	0.042088	0.704
	Group 2	18	0.0825	0.034949	

Abbreviations: GI, gingival index; PI, plaque index.

**Table 3:** Intragroup comparison of probing measurements in Group 1 at baseline and 6 months.

	Clinical parameters	*N*	Mean ± SD	Mean difference ± SD	*t*	*p* Value
1	Group 1 RH baseline	18	2.54 ± 0.5	2.48 ± 0.45	23.60	**<0.001**
	Group 1 RH 6 months	18	0.06 ± 0.13			

2	Group 1 RW baseline	18	2.87 ± 0.23	2.74 ± 0.37	31.30	**<0.001**
	Group 1 RW 6 months	18	0.13 ± 0.31			

3	Group 1 PD baseline	18	2.48 ± 0.37	0.61 ± 0.33	7.90	**<0.001**
	Group 1 PD 6 months	18	1.87 ± 0.23			

4	Group 1 CAL baseline	18	5.04 ± 0.75	3.11 ± 0.62	21.44	**<0.001**
	Group 1 CAL 6 months	18	1.93 ± 0.29			

5	Group 1 KTW baseline	18	1.96 ± 0.36	−1.06 ± 0.24	−19.00	**<0.001**
	Group 1 KTW 6 months	18	3.02 ± 0.46			

6	Group 1 GT baseline	18	1.02 ± 0.08	−0.22 ± 0.26	−3.69	**0.002**
	Group 1 GT 6 months	18	1.24 ± 0.25			

*Note*: Bold represents *p* values are statistically significant.

Abbreviations: CAL, clinical attachment level; GT, gingival thickness; KTW, keratinized tissue width; PD, probing depth; RH, recession height; RW, recession width.

**Table 4:** Intragroup comparison of probing measurements in Group 2 at baseline and 6 months.

	Clinical parameters	*N*	Mean ± SD	Mean difference ± SD	*t*	*p* Value
1.	Group 2 RH baseline	18	2.52 ± 0.51	2.44 ± 0.51	20.28	**<0.001**
	Group 2 RH 6 months	18	0.07 ± 0.14			

2.	Group 2 RW baseline	18	2.89 ± 0.23	2.69 ± 0.53	21.50	**<0.001**
	Group 2 RW 6 months	18	0.2 ± 0.4			

3.	Group 2 PD baseline	18	2.44 ± 0.38	0.57 ± 0.36	6.80	**<0.001**
	Group 2 PD 6 months	18	1.87 ± 0.23			

4.	Group 2 CAL baseline	18	4.98 ± 0.79	3.04 ± 0.73	17.62	**<0.001**
	Group 2 CAL 6 months	18	1.94 ± 0.31			

5.	Group 2 KTW baseline	18	1.94 ± 0.37	−1.07 ± 0.22	−21.14	**<0.001**
	Group 2 KTW 6 months	18	3.02 ± 0.46			

6.	Group 2 GT baseline	18	1.02 ± 0.08	−0.2 ± 0.26	−3.34	**0.004**
	Group 2 GT 6 months	18	1.22 ± 0.26			

*Note*: Bold represents *p* values are statistically significant.

Abbreviations: CAL, clinical attachment level; GT, gingival thickness; KTW, keratinized tissue width; PD, probing depth; RH, recession height; RW, recession width.

## Data Availability

The data that supports the findings of this study are available from the corresponding author upon reasonable request.
